# Chaihu-Shugan-San ameliorates tumor growth in prostate cancer promoted by depression *via* modulating sphingolipid and glycerinphospholipid metabolism

**DOI:** 10.3389/fphar.2022.1011450

**Published:** 2022-12-05

**Authors:** Wei Li, Runze Zhou, Jie Zheng, Bo Sun, Xin Jin, Min Hong, Ruini Chen

**Affiliations:** ^1^ Jiangsu Key Laboratory for Pharmacology and Safety Evaluation of Chinese Materia Medica, School of Pharmacy, Nanjing University of Chinese Medicine, Nanjing, China; ^2^ Institute of TCM-Related Comorbid Depression, School of Chinese Medicine, School of Integrated Chinese and Western Medicine, Nanjing University of Chinese Medicine, Nanjing, China; ^3^ School of Medicine and Holistic Medicine, Nanjing University of Chinese Medicine, Nanjing, China; ^4^ Department of Pharmacy, The Affiliated Suzhou Hospital of Nanjing Medical University, Suzhou Municipal Hospital, Suzhou, China

**Keywords:** Chaihu-Shugan-San, prostate cancer, depression, sphingolipid, glycerinphospholipid

## Abstract

**Background:** Psychologic depression is a pivotal pathological characteristic and has been shown to promote prostate cancer (PCa) progression. Chaihu-Shugan-San (CSS), a well-known Chinese herbal decoction, exhibits efficacy in the treatment of stress-accelerated PCa. However, the underlying mechanism of CSS in resisting PCa growth is still unknown, and further study is needed.

**Objective:** To evaluate the effects of CSS on stress-accelerated PCa in a BALB/C nude mice model and to investigate the underlying mechanisms.

**Methods:** PC-3 cells were implanted into BALB/C nude mice, and the stressed mice were exposed to chronic unpredictable mild stress (CUMS) to study the effects of CSS. The PCa growth were evaluated by tumor volume and tumor weight. Analyses of depression-like behaviors were evaluated by sucrose consumption test, tail suspension test and open field test. Network pharmacology was used to analyze the potential targets and signaling pathways of CSS against PCa. Untargeted lipidomics were used to analyze the serum lipid profiles and further elucidate the possible mechanism.

**Results:** In the CUMS stressed PCa mice, CSS can restrain tumor growth with reduced tumor volume and tumor weight, and depression-like behaviors with increased sucrose consumption, reduced immobility duration, and increased total distance and center distance. Network pharmacology suggested that the lipid metabolism-related pathways are the most likely potential targets of CSS against PCa. Using untargeted lipidomics analysis, 62 lipids were found to have significant changes in PCa mice under CUMS treatment. The levels of glycerophospholipids containing phosphatidylcholine (PC), phosphatidylethanolamine (PE), phosphatidylinositol (PI) and phosphatidylglycerol (PG), except PC (18:0_22:6) and PC (18:0_20:4), were significantly increased. Likewise, the levels of all sphingolipids (including sphingomyelin (SM), ceramides (Cer) and hexosyl-1-ceramide (Hex1Cer)) and diglyceride (DG) (32:1e) were significantly increased. CSS water extract was found to contribute to restore 32 lipids including 6 sphingolipids, 25 glycerophospholipids and 1 glyceride.

**Conclusion:** This study is the first to delineate the lipid profile of stressed PCa BALB/C nude mice using untargeted lipidomics analysis. CSS restrained tumor growth and ameliorated depression-like behaviors by reprogramming lipid metabolism. Intervention of lipid metabolism could be a preventive and therapeutic approach for PCa patients with depression.

## Introduction

PCa is one of the most commonly diagnosed cancers and the third cause of cancer-related death in men (Shen et al., 2021). Androgen deprivation therapy (ADT), the most widely used clinical treatment for PCa, results in decreased sexual function, which in turn leads to significant depression in PCa patients (Fervaha et al., 2019; Sharpley et al., 2020; Wang et al., 2020). Studies have shown that adverse emotions or psychological problems promote the development of PCa (Chang et al., 2015; Mravec et al., 2020; Eckerling et al., 2021). Growth and metastasis of cancer can be facilitated by stress responses *via* multiple ways, such as tumor microenvironment, antitumor immune activity and other indirect modulators (Wootten et al., 2015; Cheng et al., 2018; Long et al., 2021). Emerging evidence suggests that lipid metabolism is involved in psychologic depression or chronic stress through glucocorticoid pathway and inflammatory pathway (Zhou et al., 2019; Xie et al., 2020; Borsini et al., 2021). Disordered lipid metabolism is a notable feature of PCa that is driven by androgen receptor (AR) signaling (Zadra et al., 2019; Gu et al., 2021; Zhou et al., 2021). In clinical PCa, lipidomic analysis revealed higher proportions of monounsaturated phosphatidylinositol (PI) and phosphatidylserine (PS) in tumors. (Butler et al., 2021). Lipid elongation plays an important role in PCa metastasis *via* regulating protumorigenic metabolic pathway (Centenera et al., 2021; Zhou et al., 2021). The reorganization of lipid composition on the endoplasmic reticulum membrane sustains tumor-associated macrophages (TAMs) survival and pro-tumorigenic activity (Cubillos-Ruiz et al., 2016; Han et al., 2020; Di Conza et al., 2021). It is still unclear how psychologic depression modulates PCa-associated lipid metabolism.

Traditional Chinese medicine (TCM) is more and more widely used in PCa experimental treatment (Lin et al., 2019; Gai et al., 2021). The formulae, extracts and compounds of TCM have been proved to inhibit PCa progression and metastasis by various and multiple mechanisms (Wang et al., 2018; Lin et al., 2019; Gai et al., 2021; Zhang et al., 2022). In the understanding of the etiology of PCa, TCM attaches great importance to emotional factors such as “liver stagnation”, and the liver is believed to be the core of the evolution of the pathological mechanism of emotion-induced tumors. Based on the TCM theory of “Gan-zhu-shuxie” and the “Gan-axis theory” propound by Professor Huang (Zhang et al., 2011; Xie et al., 2013; Li et al., 2019), the comorbidities of depression and PCa were attributed as a result of Gan-axis dysfunction. CSS, a classical TCM Shugan formula that was first recorded in the “Yi Xue Tong Zhi” 500 years ago, is often used to treat diseases caused by liver stagnation and qi stagnation and its comorbidities, such as functional dyspepsia, cardiovascular disease and multiple tumors (Qin et al., 2013; Jia et al., 2018; Li et al., 2019; Xiao et al., 2020). Although continuous use of CSS has been reported to improve depressive phenotypes and various prostate-related diseases, the underlying mechanisms for rapid improvement in depressive symptoms and PCa treatments remain unclear.

This study is the first to demonstrate the effect of CSS on lipid metabolism reprogramming in stressed PCa mice, especially sphingolipids and glycerophospholipids, which provide new potential drug targets and key insights for PCa with psychologic depression.

## Materials and methods

### Animals and tumor implantation

BALB/c nude mice (male, 5–6 weeks old) were purchased from Changzhou Cavens Laboratory Animal Co., Ltd. (No. SCXK (SU)-2016-0010). All mice were acclimated to a standard rearing environment (Temperature: 18–22°C, Humidity: 50–60%) for 1 week before experiments carried out. For xenograft prostate tumor model, about 1 × 10^6^ PC-3 tumor cells suspended in 0.1 ml of serum-free medium were subcutaneously injected into the flank of the mice. Then tumor-bearing nude mice with CUMS treatment were randomly divided into 4 groups by weight (with 6 nude mice/group). The equivalent dose of human clinical dose of CSS as the medium dose of CSS in mice, the mice were treated with CSS in different doses (2.4 g/kg, 4.8 g/kg and 9.6 g/kg) for 6 weeks started at the time of inoculating PC-3 cells. The negative group received 0.9% normal saline. The volume of intragastric administration was 0.1 ml/10 g to each mouse. Treatments were done by oral administration at a frequency of once a day. The animals were weighed daily to adjust the gavage volume and the maximum gavage volume was no more than 0.3 ml. Tumor volume (TV) was calculated every 3 days using the following formula: TV (mm) = D/2 ×d, where D and d are the longest and the shortest diameters, respectively. At the end of the experiment, the nude mice were sacrificed, and the tumor xenografts were removed and measured. All animal experiments complied with animal ethics and all experiments were double-blind.

### CUMS treatments

CUMS was performed as described (Thaker et al., 2006; Hassan et al., 2013; Le et al., 2016). Briefly, CUMS contains several stressors including restraint (4 h), cage tilt (45°, 24 h), wet bedding (24 h), food and/or water deprivation (24 h), tail nip (1 cm from the end of the tail, 3 min), cold water swimming (4°C, 3 min), and light inversion (24 h). Stressed mice were housed singly and exposed to 3 different stressors every day. Stressed mice were restrained for 3 weeks before tumor transplantation, and continued to be restrained daily for 6 weeks.

### Reagents and drugs

MS-grade methanol, MS-grade acetonitrile, HPLC-grade 2-propanol were purchased from Thermo Fisher. HPLC-grade formic acid and HPLC-grade ammonium formate were purchased from Sigma. CSS is composed of seven herbs, including *Citrus reticulata Blanco*, *Radix Bupleuri*, *Ligusticum chuanxiong Hort*, *CyperusrotundusL.*, *Citrus aurantium L.*, *Paeonia lactiflora Pall.* and *Glycyrrhiza uralensis Fisch.*, and the ratio is 4:4:3:3:3:3:1. The herbs were purchased from the pharmacy of Jiangsu Provincial Hospital of Traditional Chinese Medicine, Nanjing University of Chinese Medicine. All herbs were authenticated by the herbal medicine botanist Professor Yunan, Zhao, Nanjing University of Chinese Medicine.

### Preparation of CSS extract

The CSS extract was prepared using a previously reported method with some modifications (Li et al., 2019). In brief, the seven crushed herbs were mixed and macerated with water at room temperature (25°C ± 2°C) and extracted by decocting twice for 30 min. After mixing the two filtrates and concentrating, the liquid medicine was frozen dry to make lyophilized powder. The yield of the extract (CSS for short) was 27.81%. The details regarding the main marker compound identifications of CSS extracts were published previously (Li et al., 2019; Liu et al., 2020).

### Behavior tests

The mice were given access to water and 1% sucrose solution at the same time. The sucrose and water intakes of every mouse were measured, and the sucrose preference rate = sucrose intake/(sucrose intake + water intake) *100%. In the tail suspension test (TST), mice were individually suspended by the distal portion of their tails with adhesive tape for 6 min. The duration of immobility was scored for the last 4 min. In the open field test (OFT), the open-field apparatus (25 cm × 25 cm × 35 cm) with 4 squares was used to assess the locomotor activity and anxiety-like behavior. Total distances and the center distances with Top Scan for 5 min were recorded as evaluation parameters. The experiment was performed 2 days before the mice were sacrificed.

### Systemic pharmacological analysis of CSS and PCa

According to the TCMSP platform, there were 174 chemical components of CSS satisfying both OB ≥ 30% and DL ≥ 0.18, which were selected as candidate active ingredients. Next, we identified the potential targets in CSS using the TCMSP platform. Moreover, known Prostate Cancer-related targets were identified from five databases by searching the key word “prostate cancer”: 1) Used the DrugBank data base and obtained 25 known targets; 2) Used the GeneCards data base and obtained 1446 known targets; 3) Used the OMIM data base and obtained 169 known targets; 4) Used the PharmGkb data base and obtained 411 known targets; 5) Used the TTD data base and obtained 92 known targets. The targets of the active components of CSS and the targets related to prostate cancer disease were fitted and screened by using the R language script. After that, we deleted the repetitive target genes, calculate drug active ingredient targets and disease targets separately to draw a “Drug_Disease” Venn diagram.

Then, we built a protein-protein interaction (PPI) network diagram by submitting the “Drug and Disease Intersection Gene” file to the STRING11.0 database. The biological species was set to “*Homo sapiens*”, the minimum interaction threshold was set to “highest confidence” (>0.95) and the rest of the settings were default settings. Based on the degree centrality (DC), betweenness centrality (BC) and closeness centrality (CC), we analyzed the network topology parameters of CSS in the treatment of PCa.

We used the R language script to enrich the “drugs and diseases intersection gene” into the Gene Ontology (GO) and Kyoto Encyclopedia of Genes and Genomes (KEGG) pathway and sum up all paths. In the GO pathway, we picked up the top 10 best recommendations for each pathway metric and in the KEGG pathway; we selected the top 30 best recommendations and maps the pathways that will be most relevant to “prostate cancer”.

### Serum sample preparation for LC-MS lipidomics study

Lipids were extracted according to MTBE method. Briefly, a 200 μL water, 800 μL MTBE and 240 μL methanol were added to 100 μL serum sample one by one and vortexed respectively. Then, the mixture was ultrasound 20 min at 4°C followed by sitting still for 30 min at room temperature. The solution was centrifuged at 14000 g for 15min at 10°C and the upper organic solvent layer was obtained and dried under nitrogen.

### LC-MS/MS for lipid analysis

Reverse phase chromatography was selected for LC separation using CSH C18 column (1.7 μm, 2.1 mm × 100 mm, Waters). The lipid extracts were re-dissolved in 200 μL 90% isopropanol/acetonitrile, centrifuged at 14000 g for 15 min, finally 3 μL of sample was injected. Solvent A was acetonitrile–water (6:4, v/v) with 0.1% formic acid and 0.1 Mm ammonium formate and solvent B was acetonitrile–isopropanol (1:9, v/v) with 0.1% formic acid and 0.1 Mm ammonium formate. The initial mobile phase was 30% solvent B at a flow rate of 300 μL/min. It was held for 2 min, and then linearly increased to 100% solvent B in 23 min, followed by equilibrating at 30% solvent B for 10 min. Mass spectra was acquired by Q-Exactive Plus in positive and negative mode, respectively. ESI parameters were optimized and preset for all measurements as follows: Source temperature, 300°C; Capillary Temp, 350°C, the ion spray voltage was set at 3000 V, S-Lens RF Level was set at 50% and the scan range of the instruments was set at m/z 200–1800. Full mass scan mode was operated for all serum samples, followed by QC identification *via* data-dependent MS/MS acquisition mode. The mass charge ratio of lipid molecules and lipid fragments was collected according to the following methods: 10 fragments (MS^2^scan, HCD) were collected after each full scan. The resolution of MS^1^ is 70,000 at m/z 200 and that of MS^2^ is 17,500 at m/z 200. Sequently contained more than 30 lipid classes and more than 1,500,000 fragment ions in the database. Both mass tolerance for precursor and fragment were set to 5 ppm.

### Data processing

LipidSearch 4.0 software was used to perform peak identification, peak extraction, and lipid identification (secondary identification) on lipid molecules and internal standard lipid molecules. The main parameters are: precursor tolerance: 5 ppm, product tolerance: 5 ppm, production threshold: 5%. According to the International Lipid Classification and Nomenclature Committee, totally 38 lipid classes and 943 lipid species were exacted from samples.

Univariate and multivariate analyses Univariate analyses including Fold Change analysis (FC) and *t*-test. Multivariate analyses including unsupervised principal component analysis (PCA), partial Least Squares discrimination analysis (PLS-DA) and orthogonal partial least squares discriminant analysis (OPLS-DA). Differential feature parameters were as follows: *p*-value < 0.05, VIP > 1.0 and FC > 1.5 or <0.67. The overlap of significantly different lipid species screened in each comparison group is displayed in the form of a Venn diagram.

To analyze the generation of annotated differentiates between lipid species (VIP>1, *p*-value < 0.05) metabolic proximities, for a deeper comprehension of how lipids interact with biological state changes. Based on the correlation analysis method, the correlation between significantly different lipids was analyzed and visually performed in the form of correlation cluster heat maps. Further, based on the lipid-lipid correlation matrix, Chord Diagrams and Network Diagrams were displayed to show the pairs of lipid species with |r|>0.8 and *p* < 0.05. Then count the difference in the content of lipid spices with different carbon chain lengths and different numbers of unsaturated bonds under each subclass.

### Statistical analysis

Statistical calculations were expressed as mean ± standard deviation (mean ± SD). Differences between groups were compared adopting one-way ANOVA analysis method by using Graphpad Prism (version 7) software, and Dunnett’s test was used for comparison between groups. A level of *p* < 0.05 was selected as the point of minimal statistical significance in every comparison.

## Results

### Depression promoted prostate tumor growth while CSS can restrain tumor growth

**FIGURE 1:**
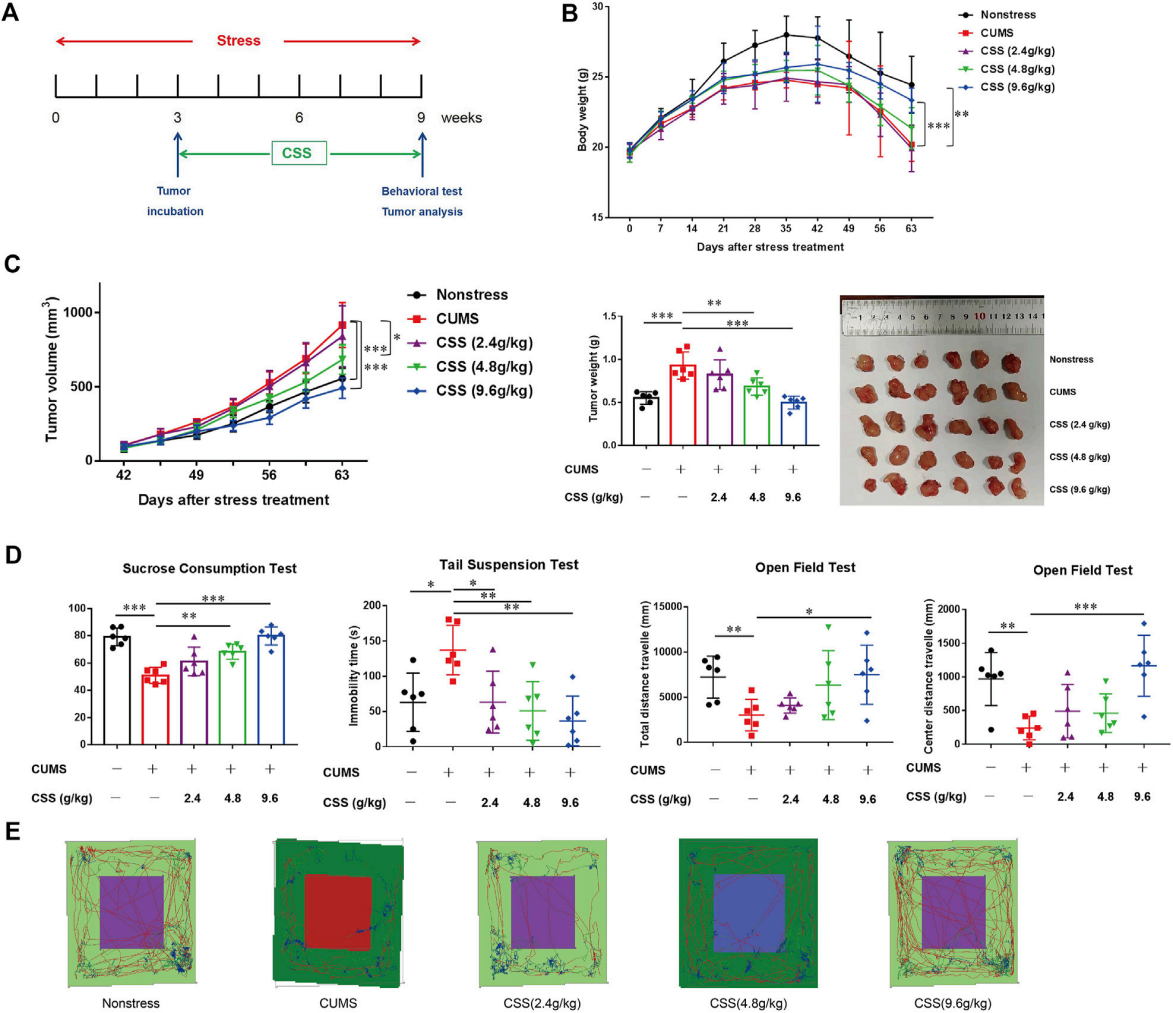
CSS prevents CUMS-promoted tumor growth in the PCa model. **(A)** Time course of restraint stress and tumor implantation. **(B)** Body weight of mice (*n* = 6). The statistical analysis was based on Two-way ANOVA (Non-stress vs. CUMS, from week 4 to week 9, **p < 0.01; CUMS vs. CSS 9.6 g/kg, from week 9, ***p < 0.001). **(C)** Tumor volume, tumor weight and tumor photo of mice (*n* = 6). The statistical analysis of tumor volume was based on Two-way ANOVA (Non-stress vs. CUMS, from week 8 to week 9, ***p < 0.001; CUMS vs. CSS 9.6 g/kg, from week 8 to week 9, ***p < 0.001; CUMS vs. CSS 4.8 g/kg, from week 8 to week 9, *p < 0.05). The statistical analysis of tumor weight was based on One-way ANOVA (**p < 0.01, ***p < 0.001). **(D)** Sucrose consumption ratio in the sucrose preference test, the immobility time in the tail suspension test, and the total distance and center distance travelled in the open field test. The statistical analysis was based on One-way ANOVA (*n* = 6; *p < 0.05, **p < 0.01 and ***p < 0.001). **(E)** The representative trace graphs in the open field test.

To test the effect of depression on PCa growth, PC-3 cells, a typical human PCa cells were implanted into BALB/C nude mice. The stressed mice were exposed to CUMS. Stressed mice were restrained for 3 weeks before tumor transplantation, and continued to be restrained daily for 6 weeks (Figure 1A). And parts of them were treated with the CSS in different doses through oral administration separately (2.4 g/kg, 4.8 g/kg and 9.6 g/kg) for 6 weeks started at the time of inoculating PC-3 cells.

We found that depression significantly decrease mouse body weight (*p* < 0.001, Figure 1B), and significantly increased tumor volume and tumor weight (*p* < 0.001, Figure 1C). And in the CSS treated groups, CSS (4.8 g/kg) and CSS (9.6 g/kg) can reduce the tumor volume and tumor weight to varying degrees (*p* < 0.05, *p* < 0.001, Figure 1C), the CSS (9.6 g/kg) even can increased the body weight of the mice compared to model (*p* < 0.05, Figure 1B).

We evaluated potential depressive-like behaviors in these mice using SPT, TST and OFT. Stressed mice exhibited less preference to sucrose in SPT trials (*p* < 0.0001; Figure 1D), and such kind of phenomenon can be improved in the CSS (4.8 g/kg) and CSS (9.6 g/kg) groups (*p* < 0.01, *p* < 0.0001 respectively; Figure 1D). Stressed mice showed a significant increase in immobility duration during TST compared to non-stressed mice (*p* < 0.01; Figure 1D), while all the CSS groups mice show significant improvement compared to CUMS mice (*p* < 0.05, *p* < 0.01, *p* < 0.01 respectively; Figure 1D). Besides, in the OFT, the stressed mice exhibited reduced the total distance and center distance, shorten the center areas stay times and the times through the center areas, but the CSS (9.6 g/kg) can improve these behaviors, increase distances and times the animals moved (*p* < 0.05; *p* < 0.001 respectively; Figures 1D,E). These results manifested those mice with CSS can reduce the depressive-like phenotype of the CUMS stressed mice and the reduction may display a tendency of dose-reliance.

### Potential antitumor mechanisms of CSS for prostate cancer

#### Screening for chemical ingredients and potential targets of CSS

We obtain 174 potential active compounds from the seven herbs that constitute CSS according to the TCMSP platform (OB ≥ 30%, DL ≥ 0.18). Then, according to these active compounds, we explored the therapeutic targets of CSS in Uniport. A total of 239 potential therapeutic targets were identified about 174 active compounds from CSS and generated the compound putative target network for CSS.

#### Target prediction of the active ingredients of CSS and PCa

**FIGURE 2:**
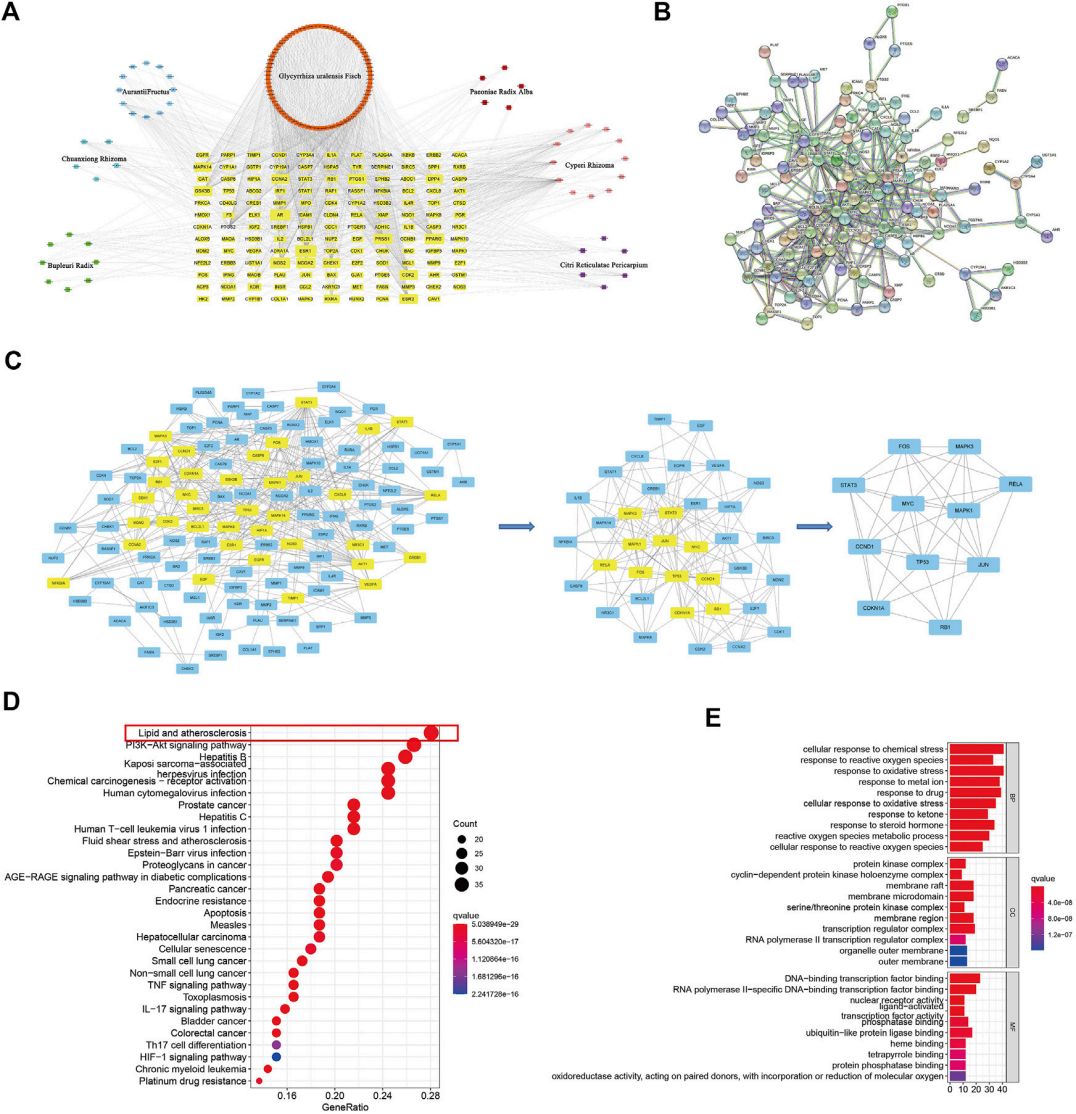
Network pharmacology screened the potential ingredients and the targets of CSS against PCa. **(A)** Dispersion map of component-target of CSS. Square represents active components of each Chinese materia medica; Yellow node represents target of chemical composition action; Each side represents interaction between the molecule of compounds and targets; There were 142 common targets. **(B)** The PPI network of the CSS compounds and PCa-related targets from this 142 common targets. **(C)** The twice analysis of PPI network got 11 nodes and 82 edges. **(D)** The KEGG pathways analysis and **(E)** GO enrichment.

We identified 1801 PCa-related targets from five databases, DrugBank, GeneCards, OMIM, PharmGkb and TTD. Then, we fitted and screened the targets of the active components of CSS and the targets related to PCa disease, which found out that there were 142 common targets (Figure 2A). Then, we constructed a PPI network of the CSS compounds and PCa-related targets from these 142 common targets (Figure 2B), which got 11 nodes and 82 edges after twice analysis. (The first analysis was based on the values of DC: 5, BC:29.97966555, CC:0.08583691 and get 36 nodes and 173 edges. The second times analysis was based on the values of DC: 8, BC:10.3092314545, CC:0.534382284 and get 11 nodes and 82 edges) (Figure 2C).

#### KEGG pathways analysis and Gene Ontology enrichment

GO and KEGG pathway results indicated that 11 significant potential targets of CSS may paiticipate in a variety of tumors, including PCa, lung cancer, bladder cancer, pancreas cancer, hepatocellular carcinoma, colorectal cancer. Moreover, the lipid and atherosclerosis, PI3K-Akt signaling pathway, IL-17signaling pathway, TNF signaling pathway, proteoglycans in cancer were enriched (Figure 2D). Besides, the results of biological process analysis showed that overlapping targets were mainly enriched in the cellular response to chemical stress, response to reactive oxygen species, response to oxidative stride, response to metal ion, response to drug, cellular response to oxidative stress, response to ketone, response to steroid hormone, reactive oxygen species metabolic process and cellular response to response reactive oxygen species (Figure 2E). Based on this, we can conclude that CSS may have therapeutic effects on PCa through 11 targets and multiple pathways. What’s more, the most likely pathway taken by CSS is lipid metabolism pathway.

#### Lipidomics study of CSS in the treatment of PCa

In this study, the lipidomics analysis were performed in three groups which were named as below: 1, Control, represents the tumor-bearing mice group without both CUMS and CSS; 2, Model, represents the tumor-bearing mice group only with CUMS; 3, CSS, represents the tumor-bearing mice group with both CUMS and CSS (9.6 g/kg).

#### Ion analyses revealed the composition and change of lipid class

**FIGURE 3:**
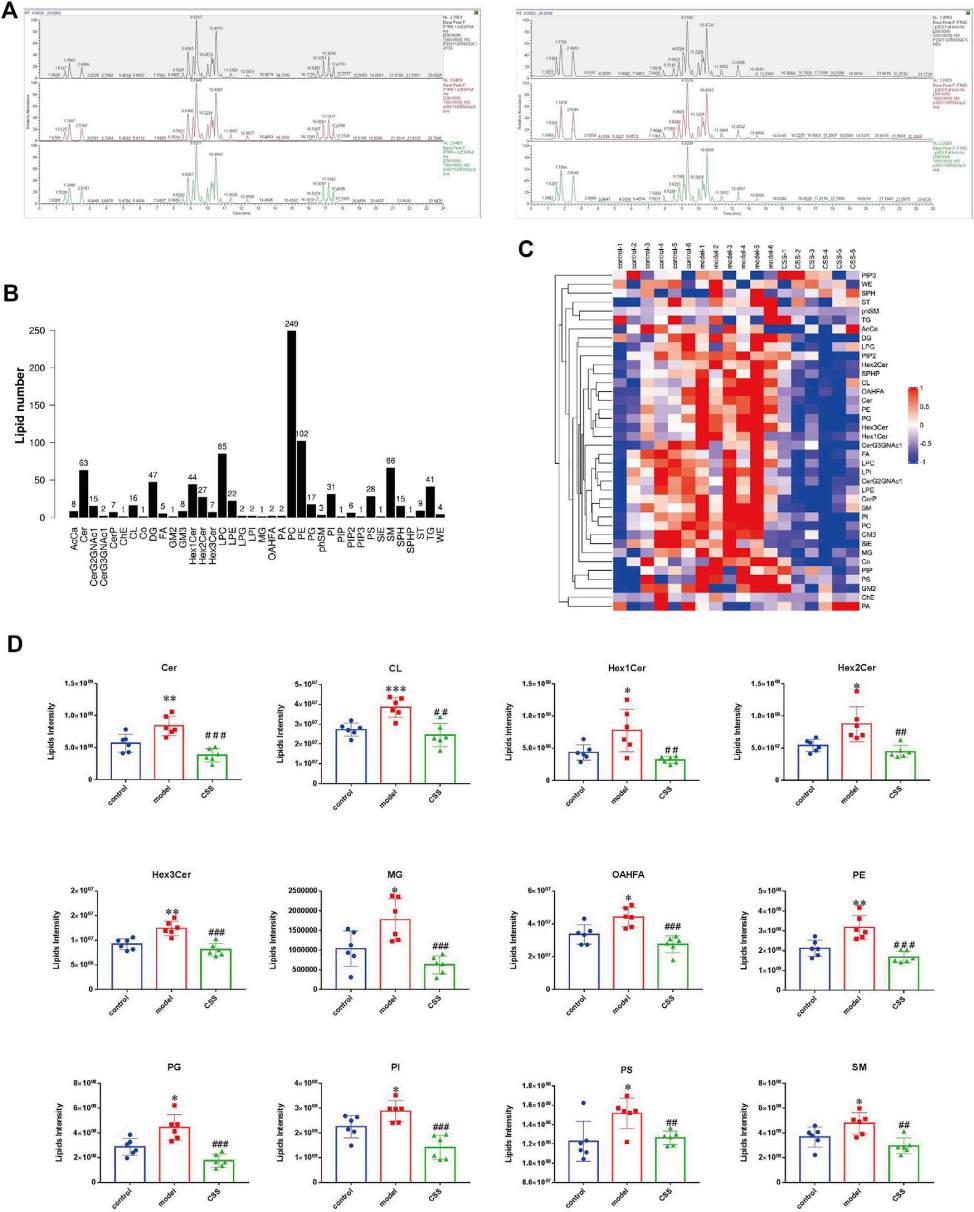
Ion analyses revealed the composition and change of lipid class. **(A)** The Total ion chromatograms (TIC) under positive and negative ion modes. Metabolomics showed very stable performance as chromatograms were anastomotic in positive ion and negative ion models. **(B)** Lipid subgroup and lipid molecule count according to the International Lipid Classification and Nomenclature Committee. **(C)** Different consistence of lipid class in control, model and CSS groups. **(D)** The 12 lipid classes with opposite trends in model vs. control (*p* < 0.05) and CSS vs. model (*p* < 0.05). AcCa, Acyl Carnitine; Cer, Ceramides; CerG2GNAc1, N-acetylhexosyl ceramide; CerG3GNAc1, Dihexosyl N-acetylhexosyl ceramide; CerP, Ceramides phosphate; ChE, Cholesterol Ester; CL, Cardiolipin; Co., Coenzyme Q; DG, Diglyceride; FA, Fatty Acid; GM2, Ganglioside, monosialo dihexosyl ceramide; GM3, Ganglioside, monosialo trihexosyl ceramide; Hex1Cer/Hex2Cer/Hex3Cer, Hexosyl ceramide; LPC, Lysophosphatidylcholine; LPE, lysophosphatidylethanolamine; LPG, Lysophosphatidylglycerol; LPI, Lysophosphatidylinositol; MG, Monoglyceride; OAHFA, (O-acyl)-1-hydroxy fatty acid; PA, Phosphatidic Acid; PC, Phosphatidylcholine; PE, Phosphatidylethanolamine; PG, Phosphatidylglycerol; phSM, Phytosphingosine; PI, Phosphatidylinositol; PIP, Phosphatidylinositol (4) phosphate; PIP2, Phosphatidylinositol (4,5) bisphosphate; PIP3, Phosphatidylinositol (3,4,5) triphosphate; PS, Phosphatidylserine; SiE, Sitosterol ester; SM, Sphingomyelin; SPH, Sphingosine; SPHP, Sphingosine phosphate; ST, Sulfatide (galactosyl ceramide sulfate); TG, Triglyceride; WE, Wax exters.

The total ion chromatograms (TIC) under positive and negative ion modes were shown in Figure 3A. Metabolomics showed very stable performance as chromatograms were anastomotic in positive ion and negative ion models. The total lipid class and lipid species population statistics of ESI^+^ and ESI^−^ for the samples were shown in Figure 3B, including 38 lipid classes and 943 lipid species. Different consistencies of lipid class in control, model and CSS groups were displayed in Figure 3C, which indicated the proportion class of different groups. Figure 3D showed the results of screening of lipid classes with opposite trends in model vs. control (*p* < 0.05) and CSS vs. model (*p* < 0.05). From the lipid composition analysis, we know that the regulation of 12 lipid classes metabolism may play a key role in the treatment of PCa by CSS.

#### Univariate and multivariate analyses of lipidomics method

**FIGURE 4:**
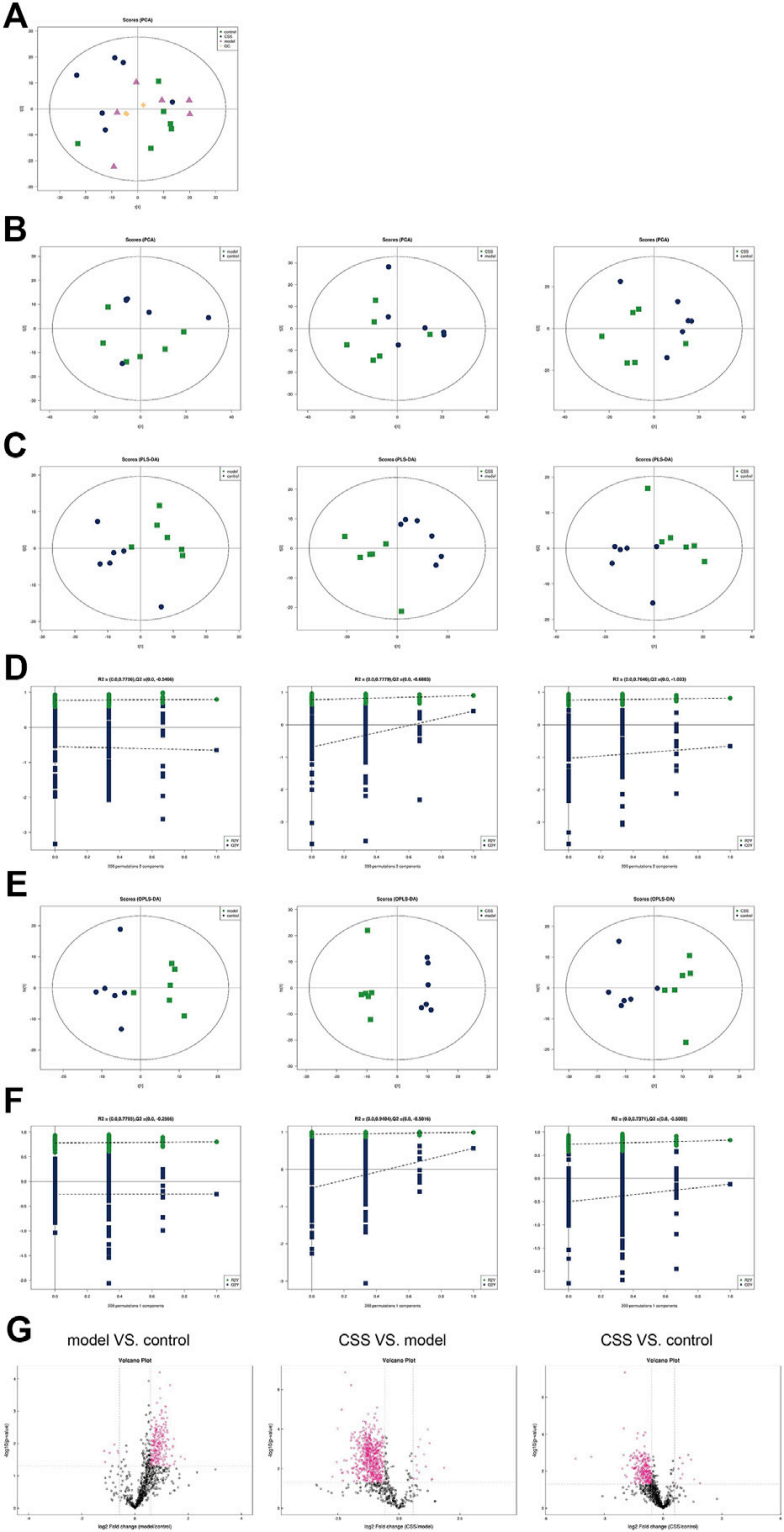
Univariate and multivariate analyses revealed stability and reliability of lipidomics method. **(A)** PCA score plot of all samples and QC samples. **(B)** PCA score plot **(C)** PLS-DA score plot **(D)** PLS-DA displacement test. **(E)** PLS-DA score plot and **(F)** OPLS-DA displacement test of model-control, CSS-model and CSS-control. **(G)** The volcano plots of model VS. control, CSS VS. model and CSS VS. control, red point represents the different lipids (FC > 2.0, *p*-value < 0.05).

The QC samples were in the middle of the three groups closely, which means the repeatability of the test (Figure 4A). In the PCA model, the scores of the PC1 and PC2 in pairs of the three groups were showed in the scores plot (Figure 4B). No clear separation can be observed between pairwise analysis of three groups from the PCA model. In the PLS-DA model, samples of model vs. control, CSS vs. model and CSS vs. control were obviously separated, indicating a significant change in lipid metabolism as a result of CUMS in PCa mice and CSS can block this change (Figure 4C). In the PLS-DA scores plot, the PC1 and PC2 showed the R^2^X, R^2^Y and Q^2^ between model vs. control and CSS vs. model (Figure 4D). In the OPLS-DA model, the differential lipids were screened based on VIP > 1.0 (Figures 4E,F). According to the VIP from OPLS-DA model, FC > 2.0 and *p* < 0.05 metabolites in model vs. control, CSS vs. model and CSS vs. control were show in Figure 4G).

#### The panel of differentially expressed lipids and correlation analysis

**FIGURE 5:**
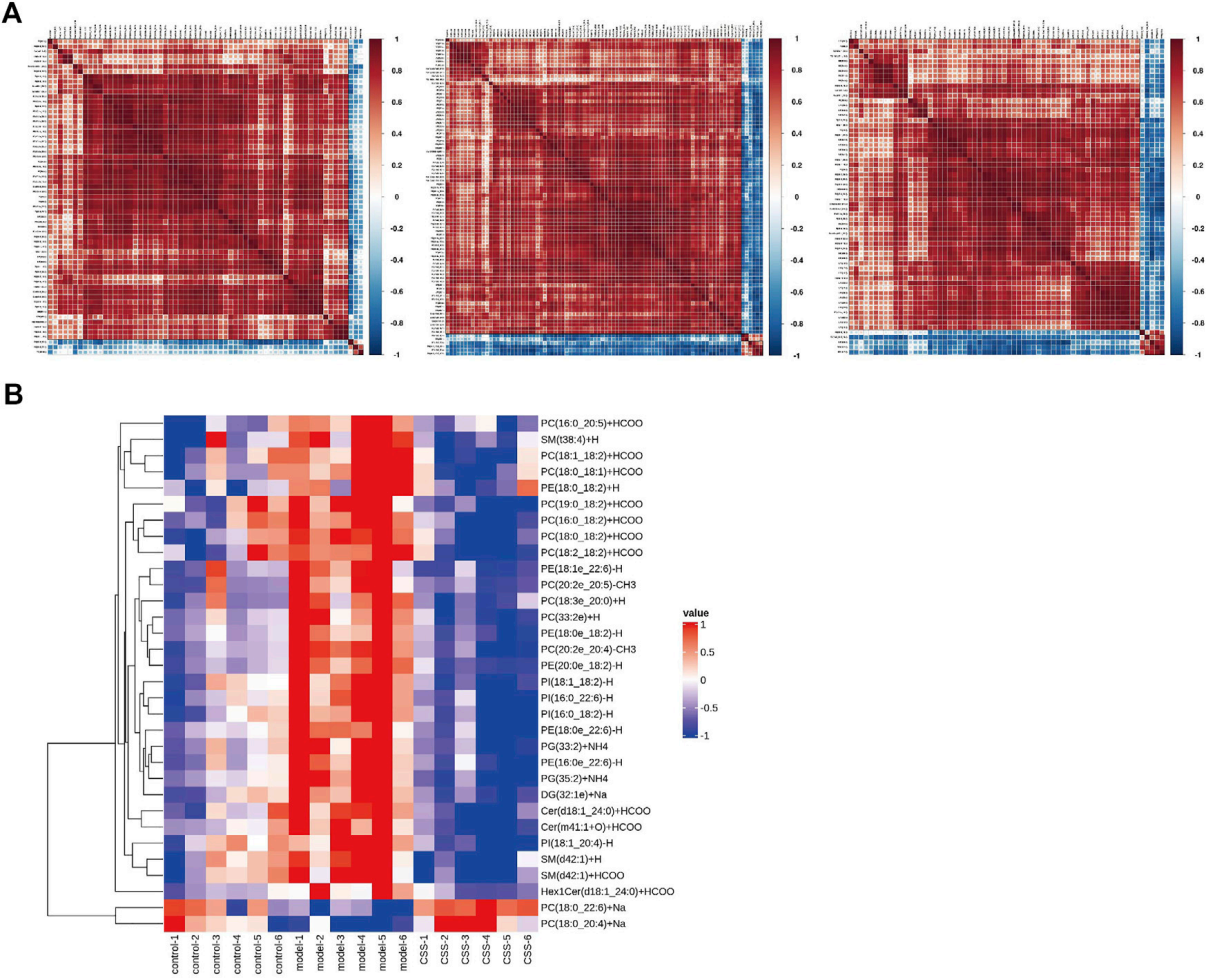
Heatmap visualization and hierarchical clustering analysis **(A)** and the correlation analysis results **(B)** based on significant difference in lipids.

**FIGURE 6:**
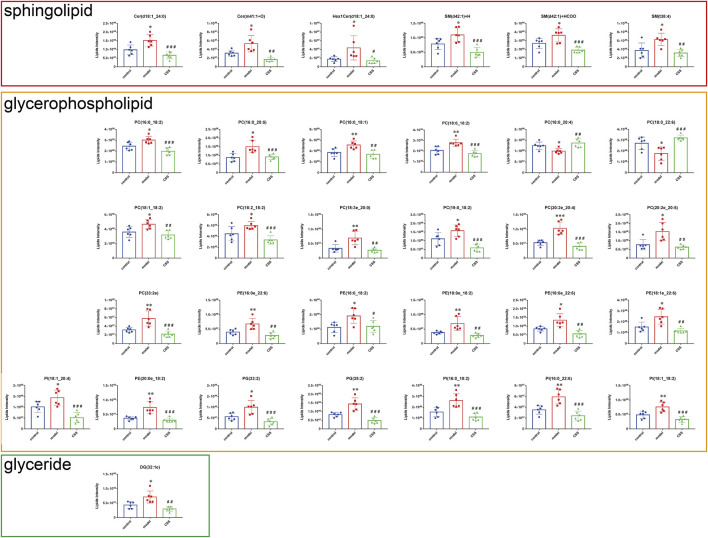
Differential expression levels (mean) of 32 differential lipids in different groups. A comparison of the relative intensities of the potential biomarkers in the control, model and CSS groups. (*p < 0.05, **p < 0.01 and ***p < 0.001 compared with control group; ^#^ *p* < 0.05, ^##^ *p* < 0.01 and ^###^ *p* < 0.001 compared with model group).

62 lipids were significantly altered in the serums of the control and model mice (VIP > 1 and *p* < 0.05). 32 lipids including 6 sphingolipids and 25 glycerophospholipids showed significant recovery after CSS treatment. As shown in Table 1, the levels of glycerophospholipids (PC, PE, PI and PG), SMs (SM, Cer and Hex1Cer) and DG (32:1e) were increased significantly in model groups. Figure 5A showed that control, model and CSS can be divided into two parts. Figure 5B showed the correlation of 32 different lipid molecules in the three groups. After CSS treatment, all lipids which changed significantly in the model group could almost return to normal levels (Figure 6). To sum up the above, CSS could regulate the lipids metabolism especially 6 sphingolipids and 25 glycerophospholipids, therefore, CSS are involved in sphingolipid metabolism and glycerophospholipid metabolism.

**TABLE 1:** Differences in lipids between control, model and CSS group.

Name	LipidIon	IonFormula	CalMz	RT-(min)	Fold Change	*p*-value	VIP	Fold Change	*p*-value	VIP
					model vs. control			CSS vs. model		
					1187pos	PC(18:0_22:6)+Na	C48 H84 O8 N1 P1 Na1	856.5826785	599.022	0.649989816	0.019610703	3.65862782	1.822240822	0.000328105	5.081411257
1071pos	PC(18:0_20:4)+Na	C46 H84 O8 N1 P1 Na1	832.5826785	618.260	0.795456453	0.011723484	2.087214027	1.378225843	0.002304993	2.972274596
1063pos	PC(18:3e_20:0)+H	C46 H89 O7 N1 P1	798.6371185	688.446	2.071364051	0.007371584	1.834342685	0.391679293	0.001555194	1.20645118
820pos	PC(33:2e)+H	C41 H81 O7 N1 P1	730.5745185	693.558	1.83659597	0.006046994	1.549763276	0.375124355	0.000968332	1.143363983
612neg	PC(19:0_18:2)+HCOO	C46 H87 O10 N1 P1	844.6073105	663.067	1.42435895	0.043685644	1.361697597	0.372188171	0.000112889	2.144248152
598neg	PC(16:0_20:5)+HCOO	C45 H79 O10 N1 P1	824.5447105	498.280	1.712238461	0.004967319	2.165652471	0.604886598	0.003999825	1.087980882
584neg	PC(18:2_18:2)+HCOO	C45 H81 O10 N1 P1	826.5603605	509.939	1.331308437	0.036792777	1.301057044	0.560873764	0.000138018	1.670421461
580neg	PC(18:1_18:2)+HCOO	C45 H83 O10 N1 P1	828.5760105	565.840	1.299460848	0.020613899	1.290421564	0.683662165	0.001575227	2.553617723
560neg	PC(18:0_18:2)+HCOO	C45 H85 O10 N1 P1	830.5916605	628.363	1.351536224	0.004964417	3.192124128	0.633762223	0.000282728	4.963877643
737neg	PC(20:2e_20:5)-CH3	C47 H81 O7 N1 P1	802.5756155	717.343	1.995546851	0.010475551	2.465679316	0.415297677	0.002012176	1.601116085
727neg	PC(20:2e_20:4)-CH3	C47 H83 O7 N1 P1	804.5912655	728.259	1.939012565	0.000399224	2.502208542	0.393605984	9.80122E-05	1.692823296
555neg	PC(18:0_18:1)+HCOO	C45 H87 O10 N1 P1	832.6073105	680.721	1.376108828	0.007591001	1.487104283	0.661381679	0.002192611	2.278146945
510neg	PC(16:0_18:2)+HCOO	C43 H81 O10 N1 P1	802.5603605	558.165	1.229819451	0.019829948	2.367153346	0.654831689	0.000279245	5.859778864
244neg	Hex1Cer(d18:1_24:0)+HCOO	C49 H94 O10 N1	856.6883225	829.889	2.482676528	0.048943233	1.621892542	0.317962469	0.029372571	1.115564404
97neg	Cer(d18:1_24:0)+HCOO	C43 H84 O5 N1	694.6354975	888.241	1.557111539	0.011691411	1.638992267	0.434078793	0.000286062	1.867815169
121neg	Cer(m41:1+O)+HCOO	C42 H82 O5 N1	680.6198475	860.580	1.694367419	0.024382626	1.210806214	0.314508028	0.001064047	1.413636465
937neg	PE(18:1e_22:6)-H	C45 H77 O7 N1 P1	774.5443155	649.534	1.619267327	0.017494697	2.252153665	0.472183948	0.001221157	1.343056541
1360pos	PE(18:0_18:2)+H	C41 H79 O8 N1 P1	744.5537835	648.354	1.71177018	0.010008795	2.583524093	0.621095538	0.021074544	1.058819575
859neg	PE(18:0e_18:2)-H	C41 H79 O7 N1 P1	728.5599655	693.112	1.829297222	0.009747283	1.511405991	0.402775049	0.002176883	1.116516566
931neg	PE(18:0e_22:6)-H	C45 H79 O7 N1 P1	776.5599655	660.837	1.5954795	0.011232552	1.796840124	0.411868829	0.001045544	1.496862666
907neg	PE(16:0e_22:6)-H	C43 H75 O7 N1 P1	748.5286655	590.798	1.70239174	0.008549525	1.410072936	0.410823488	0.001191895	1.041612108
887neg	PE(20:0e_18:2)-H	C43 H83 O7 N1 P1	756.5912655	759.107	2.002887602	0.001688457	2.097048584	0.407095123	0.000589836	1.341523909
1858pos	SM(t38:4)+H	C43 H82 O7 N2 P1	769.5854175	589.194	1.664956281	0.020401415	4.612218144	0.505181341	0.001030724	2.883641595
1807pos	SM(d42:1)+H	C47 H96 O6 N2 P1	815.7000525	808.201	1.395423421	0.035435013	4.389037487	0.450962097	0.000503958	4.079321991
1160neg	SM(d42:1)+HCOO	C48 H96 O8 N2 P1	859.6909795	807.902	1.343540309	0.041808421	1.21917041	0.530003208	0.000549102	1.227981122
1531pos	PG(35:2)+NH4	C41 H81 O10 N1 P1	778.5592635	660.945	1.745050227	0.002572905	2.658964522	0.335941001	0.000137138	2.083977283
1510pos	PG(33:2)+NH4	C39 H77 O10 N1 P1	750.5279635	591.345	1.769118954	0.012197694	1.996126019	0.330913214	0.000799377	1.666945553
1052neg	PI(16:0_22:6)-H	C47 H78 O13 N0 P1	881.5185565	455.389	1.682482653	0.0033176	1.526083297	0.408354211	0.000273271	1.075519433
1048neg	PI(18:1_20:4)-H	C47 H80 O13 N0 P1	883.5342065	481.798	1.415285028	0.039999875	1.073762162	0.359433408	0.000325287	2.105028423
1026neg	PI(18:1_18:2)-H	C45 H80 O13 N0 P1	859.5342065	491.820	1.564799912	0.00842184	1.226419794	0.428366864	0.000257296	1.223900327
1012neg	PI(16:0_18:2)-H	C43 H78 O13 N0 P1	833.5185565	483.555	1.682308076	0.007150389	3.092225609	0.418391864	0.000408211	2.253407301
304pos	DG(32:1e)+Na	C35 H68 O4 Na1	575.5009815	483.788	1.640890952	0.016426231	1.642266263	0.419176389	0.001045392	1.085013101

## Discussion

Since lipid metabolism can indicate many significant pathways, lipidomics has recently developed as new foci of PCa research. In part due to their energy storing capacity and their role as basic building blocks for cell growth, lipids are pivotal for the proliferation of cancer cells (Boroughs and DeBerardinis, 2015; Snaebjornsson et al., 2020; Bian et al., 2021). Dysregulation of signaling pathways can occur when lipid metabolism is perturbed (Bader and McGuire, 2020; Butler et al., 2021; Zhou et al., 2021). There has been research demonstrating that lipids and lipid derivatives may act as potential biomarkers of various cancers. Phosphate esters such as PC and PE are the main components of cell membranes, and changes in their chain length can directly cause changes in membrane fluidity, which in turn affects membrane permeability, material transport, and the localization of membrane proteins (Chen et al., 2018; Galbraith et al., 2018; Di Mitri et al., 2019; Watt et al., 2019; Blomme et al., 2020; Centenera et al., 2021). Sphingolipids are also involved in cancer cell biology at multiple levels (Furuya et al., 2011; Costa-Pinheiro et al., 2020; Vykoukal et al., 2020; Lin et al., 2021a; Lin et al., 2021b).

The effect of CUMS on lipid metabolism in tumor-bearing mice was first time discovered in this study by comparing the expression of differential lipids. There were significant differences in the levels of 62 lipids between the serums of control and model mice, and provided potential clinical aims for diagnosis or prognosis. In addition, we observed significant changes in 6 sphingolipids, 1 glyceride and 25 glycerophospholipids by CSS, so we speculate that its lipid changes may be related to the therapeutic effect of CSS on PCa combined with depression.

### Change of sphingolipids metabolism

In this study, we observed that the levels of 6 Sphingolipids (including Cer(d18:1_24:0) +HCOO, Cer(m41:1+O) +HCOO, Hex1Cer(d18:1_24:0) +HCOO, SM(t38:4) +H, SM(d42:1) +H and SM(d42:1) +HCOO) were display the annotated differential metabolites in model vs. control and CSS vs. model. Cer is the central molecule in all sphingomyelin metabolism. Sphingomyelin is produced by ceramide and phosphatidylcholine, and the reaction is catalyzed by sphingomyelin synthase (Morigny et al., 2020). Stress response, differentiation, proliferation, apoptosis and migration of tumor cells are all regulated by sphingolipids receptor, especially Cer, sphingosine-1-phosphate (S1P) and ceramide-1-phosphate (C1P) (Pchejetski et al., 2008; Guillermet-Guibert et al., 2009; Huang et al., 2013; Gomez-Larrauri et al., 2020; Presa et al., 2020; Camacho et al., 2022). What’s more, sphingolipids are the most important elements of the lipid raft. Key signals for survival and progression of PCa cells are transmitted through the lipid raft. SM and cholesterol can regulate the dynamics of the lipid raft to affect important signaling pathways such as EFGR and Akt pathways to maintain PCa cells survival and growth (Abe and Kobayashi, 2017). Thus, SM could influence PCa growth and malignancy progression by interfering with lipid rafts.

### Change of glycerophospholipid metabolism

The 40–50% proportion of the membranes in living organisms is composed of PC, which is one of the most abundant phospholipids. (van der Veen et al., 2017). PC is one of the major constituents of cell membranes and lipid signaling, and plays an important role in cell membrane signaling and enzyme activation (Blom et al., 2011). In carcinomas, it is particularly important for the maintenance of membrane biosynthetic value-added in cancer cells. Furthermore, Kanphorst et al. found that extracellular phospholipids are downregulated in cells under hypoxia due to lipid clearance (Kamphorst et al., 2015). Thus, in most human cancer cells under hypoxia, the activity of the oxygen-dependent enzyme stearoyl-CoA desaturase (SCD)1 is well reduced and fails to promote lipogenesis, which can lead to a deficiency of fatty acids in cancer cells (Luis et al., 2021). Tumors need to consume large amounts of phospholipids for cancer cell proliferation, which may be an important level of reduced phospholipid levels and thus higher PC and LPC consumption rates in cancer cells compared to normal cells (Kopecka et al., 2020). At the same time, we found that LPC may have a serious impact on the promotion of inflammation generation during PC degradation. In the lipidomic analysis, we observed a significant increase in PC and LPC concentrations in model vs. control and CSS can reverse this change. Several previous studies have reported similar results regarding phospholipid levels in multiple cancers. As a result of these findings, CSS may be able to suppress excessive lipid metabolism in PCa under CUMS.

## Conclusion

In this study, the lipid profiles of PCa bearing BALB/c nude mice following treatment with CUMS were examined for the first time. Significant variations of 62 lipids were found in the serums lipid profiles of the control and model mice. 32 lipids including 6 sphingolipids, 25 glycerophospholipids and 1 glyceride appeared to show significant recovery after CSS treatment. CSS can therefore treat stress-accelerated PCa growth by regulating sphingolipid metabolism and glycerophospholipid metabolism. However, the potential mechanism linking lipid metabolic reprogramming with PCa still needs further experiments to verify. The effects of CUMS on lipid metabolism in tumor-bearing mice with other PCa cells (e.g., LNCaP and DU145) need further study.

## Data Availability

The raw data supporting the conclusion of this article will be made available by the authors, without undue reservation.

## Abbreviations

ADT, androgen deprivation therapy; C1P, ceramide-1-phosphate; Cer, ceramides; CRPC, hormone-resistant prostate cancer; CSS, Chaihu-Shugan-San; CUMS, chronic unpredictable mild stress; DG, diglyceride; GO, Gene Ontology; Hex1Cer, hexosyl-1-ceramide; KEGG, Kyoto Encyclopedia of Genes and Genomes; LCAT, lecithin-cholesterol acyltransferase; LPC, Lysophosphatidyl choline; OFT, open field test; PC, phosphatidylcholine; PCa, prostate cancer; PE, phosphatidylethanolamine; PG, phosphatidylglycerol; PI, phosphatidylinositol; PPI, protein-protein interaction; PS, phosphatidylserine; S1P, sphingosine-1-phosphate; SCD, stearoyl-CoA desaturase; SM, sphingomyelin; SPT, sucrose preference test; TAMs, tumor-associated macrophages; TCM, traditional Chinese medicine; TST, tail suspension test.
